# Sheng Jing Decoction Can Promote Spermatogenesis and Increase Sperm Motility of the Oligozoospermia Mouse Model

**DOI:** 10.1155/2021/3686494

**Published:** 2021-12-01

**Authors:** Guang Yan, Fang Tian, Peng Liu, Jianming Sun, Jianmin Mao, Wenjun Han, Ran Mo, Shishuai Guo, Quanyao Yu

**Affiliations:** ^1^Department of Urology and Reproductive Medicine, Seventh People's Hospital of Shanghai University of TCM, Shanghai 200137, China; ^2^NHC Key Lab of Reproduction Regulation (Shanghai Institute for Biomedical and Pharmaceutical Technologies), Fudan University, Shanghai 200032, China

## Abstract

Sheng Jing Decoction (SJD), as a traditional Chinese medicine prescription, is mainly be used to treat male infertility. However, the pharmacological functions and molecular mechanisms of SJD are poorly understood. In this study, we investigated the functions of SJD on spermatogenesis and sperm motility and explored the potential mechanisms involved. Here, we demonstrated that high, medium, and low doses of SJD are effective in restoring the impairments of the whole body and testicular tissue by cyclophosphamide inducing and to rescue the damage of testicular tissue cells including Sertoli cells and germ cells. SJD can partly restore the decrease in sperm concentration, sperm vitality, sperm motility, and normal sperm morphology rate in oligozoospermic mouse models. Ki67 staining analyses confirm SJD can promote testicular tissue cell proliferation. Real-time RT-PCR analyses also reveal that SJD can upregulate the expression of proliferation-associated gene *Lin28a* and differentiation-associated genes *Kit*, *Sohlh2*, and *Stra8*. SJD can also reduce the impairment of mitochondrial membrane potential (MMP) and sperm plasma membrane integrity by cyclophosphamide inducing. Our results reveal that SJD is effective in improving both sperm quantity and quality by increasing the sperm concentration, sperm vitality, sperm motility, and normal sperm morphology rate. SJD can promote spermatogenesis by upregulating the expression of the proliferation-associated gene *Lin28a* and the differentiation-associated genes (*Kit*, *Sohlh2,* and *Stra8*). SJD can sustain MMP and sperm plasma membrane integrity to increase sperm motility.

## 1. Introduction

Infertility is defined by the World Health Organization as a disorder of the reproductive system, characterized by failure to achieve a clinical pregnancy after ≥12 months of regular unprotected intercourse [1]. Male infertility normally refers a male's inability to cause pregnancy in a fertile female partner. Infertility affects up to 15% of couples, and male fertility has been found to be deficient in no fewer than 50% of infertile couples [2, 3]. Male infertility is a multifactorial pathological condition that affects approximately 7% of the male population [4]. The four major aetiological categories of male infertility are spermatogenic quantitative defects, ductal obstruction or dysfunction, hypothalamic-pituitary axis disturbances, and spermatogenic qualitative defects [5]. The spermatogenesis disorder caused by genetic and nongenetic factors contributes to spermatogenic quantitative and qualitative defects [6]. Spermatogenesis is a highly organized and complex process regulated by multiple genes. Spermatogonial stem cells (SSCs) and progenitor spermatogonia express self-renewal- and proliferation-associated genes such as *Pou5f1* [7], *Lin28a* [8, 9], *Mir-21* [10], *Mir-17∼92* (*Mirc1*) [11], *Foxo1* [12], *Nanos2* [13], *Neurog 3*(*Ngn3*) [14], *Sox3* [15], *Taf4b* [16], and *Zbtb16* (*Plzf*) [17] to maintain the capacity for self-renewal and proliferation. During spermatogonial differentiation, upregulated genes are associated with differentiation such as *Sohlh1* [18], *Sohlh2* [19], *Stra8* [20], *Kit* [21], *Ccnd2* [22], and *Sall4* [23] to promote SSCs to differentiate into mature haploid spermatozoa. However, evidence-based data are limited in the empirical use of drugs for the disorder of spermatogenesis, and some drugs have obvious side effects.

In China, traditional Chinese medicine (TCM) has been employed to treat male infertility for more than 2000 years, and it has been appealed increasingly used in the surrounding areas and Western countries. Holism and treatments based on syndrome differentiation are the essence and the basic characteristics of TCM, and utilizing this theory for treating male infertility yields satisfactory results [24, 25]. Sheng Jing Decoction (SJD) is one of the traditional Chinese medicine prescriptions mainly used to treat male infertility. SJD consists of several herbs, including *Rehmannia glutinosa* (Gaertn.) DC., *Astragalus membranaceus* (Fisch.) Bunge, *Pseudostellaria heterophylla* (Miq.) Pax, *Dipsacus acaulis* (A. Rich.) Napper, *Lycium arenicolum* Miers, *Astragalus complanatus* Bunge, and *Gleditsia sinensis* Lam. [26]. SJD has been applied in many hospitals in China, and patient reactions are effective. However, the mechanism of action underlying the therapeutic effects of SJD is not fully understood. In this study, we used the oligozoospermia model of ICR mice to verify the function of SJD and to explore its mechanism. We found that SJD can increase the sperm concentration, sperm vitality, sperm motility, and normal sperm morphology rate. We further confirm that SJD enhances sperm vitality and motility by maintaining sperm mitochondrial function and sperm plasma membrane integrity. SJD can promote spermatogenesis by upregulating the expression of the proliferation-associated gene *Lin28a* and the differentiation-associated genes (*Kit*, *Sohlh2*, and *Stra8*).

## 2. Methods and Materials

### 2.1. Experimental Animals

Male ICR mice were purchased from the Shanghai Experimental Animal Center and kept in the Experimental Animal Building of the Shanghai Institute of Family Planning Science (Clean grade, license No. SCXY(Shanghai)2018-0017) [26].

### 2.2. Reagents and Antibodies

Cyclophosphamide (8H257F, Baxter International Inc., Illinois, USA), anti-TRA98 (ab82527, Abcam, Cambridge, MA, USA), anti-GATA4 (ab84593, Abcam, Cambridge, MA, USA), anti-3*β*-HSD (sc-515120, Santa Cruz Biotechnology, Dallas, TX, USA), and anti-Ki67 antibody (AF1738, Beyotime, Shanghai, China) were used. The TUNEL kit (C1086, Beyotime, Shanghai, China), LIVE/DEAD Sperm Viability kit (Thermo Fisher Scientific, Waltham, MA), and PSA-FITC (012M4037V, Sigma, St. Louis, MO, USA) were also used.

### 2.3. Preparation of the SJD

SJD was combined with *Rehmannia glutinosa* (Gaertn.) DC., *Astragalus membranaceus* (Fisch.) Bunge, *Pseudostellaria heterophylla* (Miq.) Pax, *Dipsacus acaulis* (A. Rich.) Napper, *Lycium arenicolum* Miers, *Astragalus complanatus* Bunge, and *Gleditsia sinensis* Lam. All herbs pieces were purchased from the Shanghai Wanshicheng TCM Co., Ltd, and made into SJD by TCM Pharmacy of Seventh People's Hospital of Shanghai University of TCM. *Rehmannia glutinosa* (Gaertn.) DC., *Astragalus membranaceus* (Fisch.) Bunge, *Dipsacus acaulis* (A. Rich.) Napper, *Lycium arenicolum* Miers, *Astragalus complanatus* Bunge, and *Gleditsia sinensis* Lam. can be checked in “The Plant List” (https://www.theplantlist.org), and *Pseudostellaria heterophylla* (Miq.) Pax is available in MPNS (https://mpns.kew.org). The details of the herbs are shown in Supplementary Table 1 [26].

### 2.4. Experimental Design

The oligozoospermia model mice were prepared by cyclophosphamide inducing. Cyclophosphamide was dissolved in PBS and injected intraperitoneally (ip) to the mice (60 mg/kg), once in a day for a period of 5 days. High (33g/kg), medium (16.5 g/kg), and low (8.25 g/kg) doses of SJD were given orally to mice, once in a day for a period of 35 days. The medium dose is converted from the therapeutic dose of humans. Body weights were measured every week. The testes were quickly dissected out and weighed. One half of the testes were collected and stored at −70°C for real-time RT-PCR analysis, and the other half of the testes were fixed in Bouin's fixative (0.2% picric acid/2% (v/v) formaldehyde in PBS) for histological evaluation [26].

### 2.5. Sperm Motility and Count

The cauda epididymis was placed in 1 mL normal saline, and then, a deep hole was cut in each caudate nucleus with a microscissors. Sperm was allowed to release for 5 minutes in an incubator with a temperature of 34°C and 5% CO_2_. The sperm suspension was incubated in a CO_2_ incubator for 30 min, and a drop of sperm suspension was uniformly smeared on a clean glass slide. Sperm motility and count were analyzed by using the CASA system (IVOII Sperm Analyzer, Hamilton Thorne Inc., Beverly, USA). The sperm suspension smear was stained with Papanicolaou. At least 200 sperms were counted to assess the abnormal sperm percentage.

### 2.6. Immunohistochemistry

Histological sections were treated with 3% (v/v) H_2_O_2_ to block endogenous peroxides, and antigen retrieval was performed in a nitrate buffer (pH 6.1) at 95°C for 30 min. Goat serum (FBS, 10% (v/v)) was used to block nonspecific staining. The sections were then incubated with the primary antibodies such as anti-TRA98 (1 : 200), anti- GATA4 (1 : 200), anti-3*β*-HSD (1 : 100), and anti- Ki67(1 : 100); the ABC Kit (Vector, USA) and DAB (ZSGB-bio, China) were used for staining. The images were observed using a bright-field microscope (BX43 Olympus, Tokyo, Japan) and captured using a color digital camera (DFC425 Leica, Wetzlar, Germany).

### 2.7. Real-Time RT-PCR Analysis

Total RNA was prepared using TRIzol (Sigma, USA) and was reversely transcribed into cDNA with the QuantiNova Reverse Transcription Kit (Qiagen, Düsseldorf, Germany). Real-time quantitative PCR was performed using a CFX96™ real-time system (Bio-Rad, USA) and SYBR Premix Ex Taq (TakaRa, Dalian, China) according to the manufacturer's instruction. Raw data were normalized to the internal *GAPDH* and presented as relative expression level calculated by the 2^−△△Ct^ method. Primer pairs were as follows: *Lin28a*:5′-GGTGGTGTGTTCTGTATTGGGA-3′ and 5′-AGTTGTAGCACCTGTCTCCCTTTG-3′; *Kit*: 5′-GGGCTAGCCAGAGACATCAG-3′ and 5′-AGGAGAAGAGCTCCCAGAGG-3′; *Sohlh2*: 5′-TCTCAGCCACATCAC AGAGG-3′ and 5′-GGGGACGCGAGTCTTATACA-3′; and *Stra8*: 5′-ACCCG GTAGGGCTCTTCAA-3′ and 5′-GACCTCCTCTAAGCTGTTGGG-3′ [26].

### 2.8. Mitochondrial Membrane Potential Assay

Mitochondrial membrane potential (MMP) was assessed using the JC-1 staining kit (Beyotime). The treated spermatozoa were stained with the JC-1 working fluid according to the manufacturer`s instructions. Microphotographs of the spermatozoa were obtained using a fluorescence microscope (BX51 Olympus), taking separate images with blue and green bandpass filters, corresponding to the green fluorescence of the JC-1 monomer (spermatozoa with low MMP) and the red fluorescence of the multimeric aggregates (spermatozoa with normal MMP), respectively. MMP collapse was indicated by an increase in the green/red fluorescence intensity ratio. Semiquantitative analysis was performed using ImageJ.

### 2.9. Sperm Plasma Membrane Integrity Test

According to the LIVE/DEAD Sperm Viability Kit image protocol (Thermo Fisher Scientific), SYBR 14 stock solution and propidium iodide(PI) solution were added to the diluted semen(1 × 10^7^/mL), with a final concentration of SYBR 14 and PI at 100 nM and 12 *μ*M, respectively. The cells were then incubated in the dark for 5 min at 37°C. After washing, the stained sperms were observed by using a fluorescence microscope (BX51 Olympus, Tokyo, Japan). Sperm plasma membrane integrity rate (%) = [(SYBR 14^+^ cells–SYBR 14^+^ PI^+^ cells) ÷ SYBR 14^+^ cells] × 100%.

### 2.10. Statistics and Data Analyses

Data are expressed as the mean ± SEM, and statistical evaluations were performed using one-way ANOVA (Tukey's multiple comparisons test). Values of *p* < 0.05 were considered statistically significant.

## 3. Results

### 3.1. SJD Can Accelerate Recovery Impairments Caused by Cyclophosphamide Inducing in the Whole Body and Testicular Tissues

**Figure 1:**
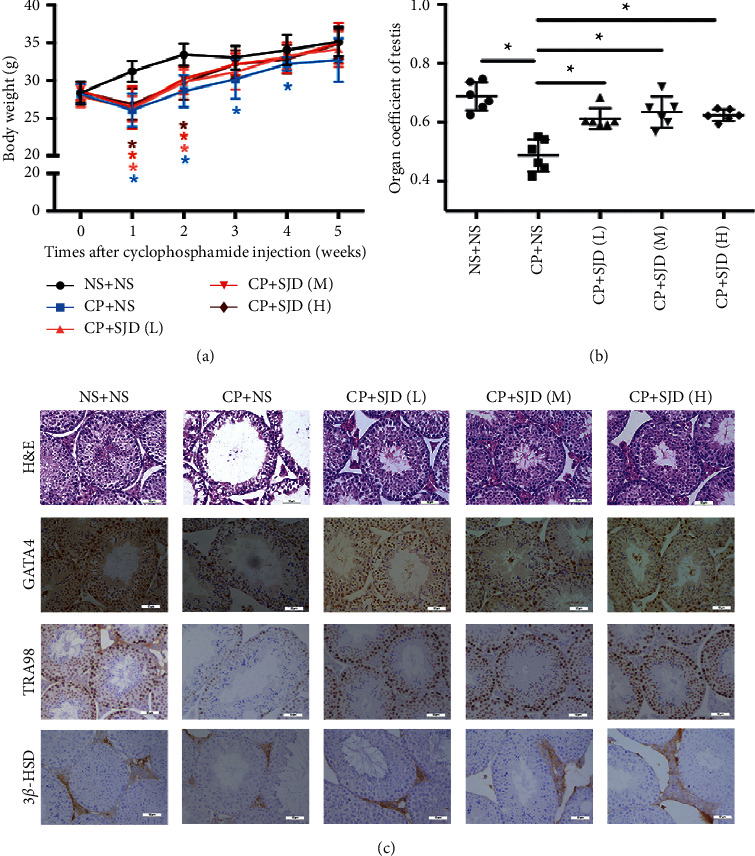
SJD promotes recovery from impairment caused by cyclophosphamide inducing. (a) Effect of cyclophosphamide and SJD treatment on the whole body. Body weights were obtained after the 1st–5th weeks of last injection. Data were means ± SEM. *n* = 6 in each group, ^*∗*^*p* < 0.05. (b) Effect of cyclophosphamide and SJD treatment on the relative weight of the testes. Data were collected after the 5th week of last injection. Data were means ± SEM. *n* = 6 in each group, ^*∗*^*p* < 0.05. (c) H&E and immunohistochemistry analysis of GATA4, TRA98, and 3*β*-HSD expression in testicular tissues. Scale bar, 50 *μ*m. NS: normal saline, CP: cyclophosphamide, SJD(L): low doses of Sheng Jing Decoction, SJD(M): medium doses of Sheng Jing Decoction, and SJD(H): high doses of Sheng Jing Decoction.

To investigate the function of SJD, we constructed an oligozoospermia model of ICR mice by ip administration of 60 mg/kg dose of cyclophosphamide [27]. Simultaneous with modeling, high, medium, and low doses of SJD were administered orally for treatment, respectively. We measured the body weight of mice after ip administration of cyclophosphamide every week and testis weight for SJD treatment for 35 days. The high, medium, and low doses of SJD can all speed up the recovery from weight loss of the whole body by cyclophosphamide inducing (Figure 1(a)). The relative weight of the testes of oligozoospermia model mice significantly increased after SJD treatment (Figure 1(b)). H&E staining of the fresh testicular tissue found that testicular tissue was severely damaged by cyclophosphamide inducing, and SJD was helpful in alleviating the pathological damage (Figure 1(c)). Testicular tissue cells mainly include Sertoli cells, germ cells, and Leydig cells, which can be determined by their respective molecular markers (GATA4, TRA98, and 3*β*-HSD). Immunohistochemistry data showed that Sertoli cells and germ cells were remarkably damaged in the oligozoospermia model mice and could be partly recovered by SJD treatment (Figure 1(c)), whereas Leydig cells showed no significant differences among the different groups (Figure 1(c)).

### 3.2. SJD Can Partly Restore the Defects of Sperm Quantity and Quality in Oligozoospermic Mouse Models

**Figure 2:**
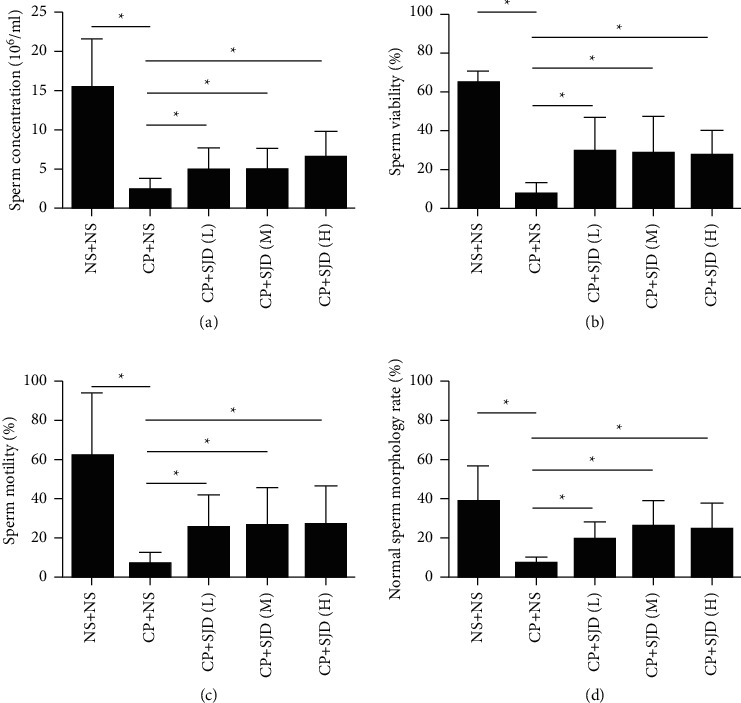
SJD increases the sperm quantity and quality of oligozoospermia model mice. (a, b, c) The concentration, vitality, and motility of sperm by computer-aided sperm analysis (CASA). (d) Normal sperm morphology rate was determined by PAP staining. Data were means ± SEM. *n* = 6 in each group, ^*∗*^*p* < 0.05.

The concentration, vitality, and motility of sperm were evaluated by computer-aided sperm analysis (CASA). CASA reports revealed that the sperm concentration, vitality, and motility of oligozoospermic mouse models were significantly decreased compared with the control group, whereas there was a significant improvement in sperm concentration, vitality, and motility after SJD treatment (Figures 2(a)–2(c)). The normal sperm morphology rate was markedly increased in oligozoospermia model mice by SJD treatment compared to that of mice without SJD treatment (Figure 2(d)).

### 3.3. SJD Can Promote Spermatogenesis and Upregulate the Expression of the Proliferation-Associated Gene Lin28a and the Differentiation-Associated Genes (Kit, Sohlh2, and Stra8)

**Figure 3:**
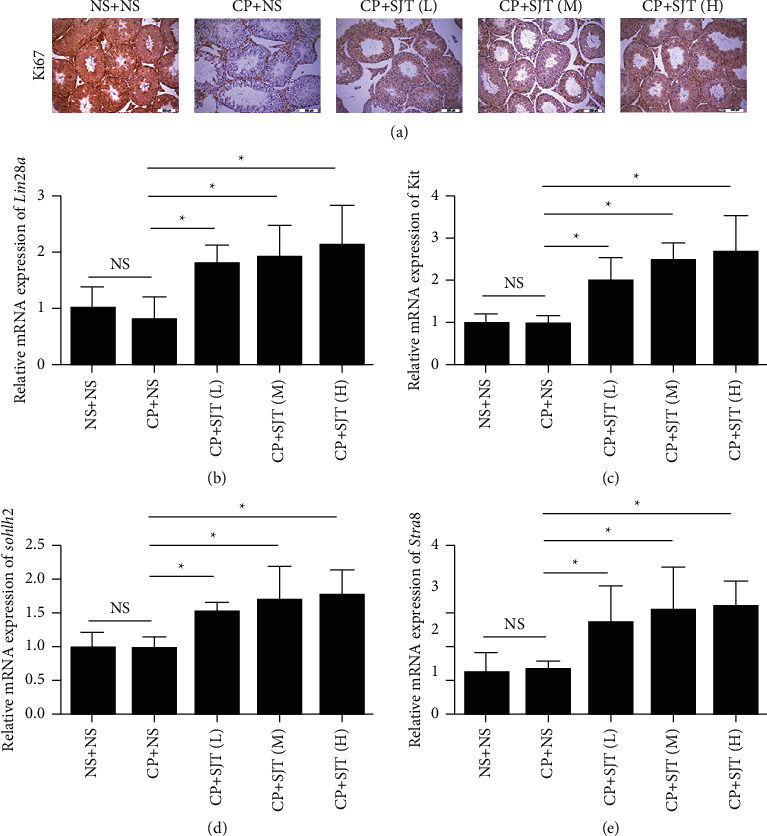
SJD promotes spermatogenesis and upregulates genes *Lin28a, Kit*, *Sohlh2,* and *Stra8.* (a) The proliferation ability of testicular tissue cells was reflected by Ki-67 staining. (b, c, d, e) Real-time RT-PCR analysis of *Lin28a, Kit*, *Sohlh2,* and *Stra8* in oligozoospermia model mice after SJD treatment and control group mice. Data were means ± SEM. *n* = 6 in each group, ^*∗*^*p* < 0.05.

The abovementioned data showed that SJD can significantly increase the number of Sertoli cells and germ cells of oligozoospermic mouse models. In addition, Ki67 staining analysis also confirmed that SJD promoted proliferation of testicular tissue cells in oligozoospermic mouse models (Figure 3(a)). To further investigate the mechanism of SJD, we examined the mRNA expression of the proliferation-associated gene *Lin28a* and the differentiation-associated genes (*Kit*, *Sohlh2,* and *Stra8*) through quantitative RT-PCR. The results suggested that the expression of genes *Lin28a, Kit*, *Sohlh2,* and *Stra8* had no difference in oligozoospermic mouse models without SJD treatment compared with those of the normal mice, but significantly upregulated in oligozoospermia model mice by SJD treatment (Figures 3(b)–3(e)).

### 3.4. SJD Has the Effect on MMP and Sperm Plasma Membrane Integrity

**Figure 4:**
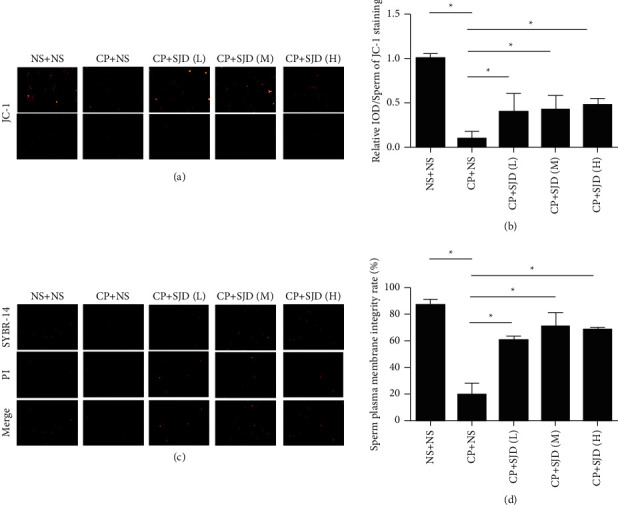
Effect of SJD treatment on sperm mitochondrial membrane potential (MMP) and sperm plasma membrane integrity. (a, b) MMP was tested and analyzed by JC-1 in oligozoospermia model mice after SJD treatment and control group mice. (c, d) Sperm plasma membrane integrity was determined by SYBR-14/PI double staining in oligozoospermia model mice after SJD treatment and control group mice. Data were means ± SEM. ^*∗*^*p* < 0.05.

Sperm MMP and sperm plasma membrane integrity were two important factors involved in sperm motility. MMP was tested and analyzed by JC-1 staining. The results showed MMP was remarkably decreased by cyclophosphamide inducing, whereas SJD treatment could offset the loss of MMP (Figures 4(a) and 4(b)). SYBR-14/PI double staining analysis showed that the sperm plasma membrane integrity was less impaired in oligozoospermic mouse models by SJD treatment compared with that in models without SJD treatment (Figures 4(c) and 4(d)).

## 4. Discussion

Male infertility in recent years has been attracting more attention from the public because of the evidence of decline in semen quality. More than 90% of male infertility cases are due to low sperm counts, poor sperm quality, or both [28, 29]. Many factors contribute to the male infertility including abnormal spermatogenesis [30, 31]; reproductive tract anomalies or obstruction [32]; inadequate sexual and ejaculatory functions [33]; impaired sperm motility [34]; imbalance in hormone levels [35]; and genetic defects [4]. In addition, 30% of infertile men suffer from idiopathic infertility [36]. Although a great progress has been made in treating male infertility, including intrauterine insemination, *in vitro* fertilization (IVF), and even intracytoplasmic sperm injection (ICSI), these treatments are sometimes ineffective, invasive, and expensive or associated with high risks. Therefore, it is necessary to seek effective natural remedies to enhance fertility. TCM has been used to improve male infertility in China for a very long time and has now become increasingly popular in its surrounding areas and Western countries for treating infertility [24, 37]. SJD, as a TCM prescription, has been used to treat male infertility in many Chinese hospitals and has helped many men with fertility problems achieve clinical pregnancy. However, its biological function and the relevant mechanisms are far from clarified [26].

Cyclophosphamide is as an anticancer drug that mainly functions to cause DNA damage and promote cell death [38–41]. The cytotoxic effect of cyclophosphamide targets rapidly divides cells, and the testis is a highly sensitive organ for damaging effects [27, 42]. Cyclophosphamide causes temporary interference of the normal male reproductive system with low-dose treatment [27]. To investigate the role of SJD in treating male infertility, we established oligozoospermia models of ICR mice by cyclophosphamide inducing. There was a remarkable impairment of sperm and its fertilizing ability in the oligozoospermic mouse models (Figure 2). The high, medium, and low doses of SJD were all helpful in restoring the damage to testicular tissues (Figure 1(c)) and increased sperm quantity and quality (Figure 2). Although the effect of SJD did not show a significant dose-dependent relationship, our data verified the positive effect of SJD on spermatogenesis. SJD is a mixed preparation with multiple active ingredients, and it is difficult to show an obvious dose-dependent curve. Establishing fingerprint and finding the key effective components of SJD is the focus of our future study.

Male fertility is maintained through intricate cellular and molecular interactions that ensure SSCs proceed in a step-wise differentiation process through spermatogenesis and spermiogenesis to produce sperm [6]. Spermatogenesis is a highly organized and complex process that allows for the continuous production of millions of haploid spermatozoa throughout the male adult life and for transferring the intact genome and appropriate epigenome through generations [31, 43]. Our data showed that the expression of the Sertoli cell marker GATA4 [44] and germ cell marker TRA98 [45] was increased by SJD treatment in oligozoospermic mouse models (Figure 1(c)) and sperm concentration and vitality were significantly increased by SJD treatment (Figures 2(a) and 2(b)). In addition, SJD enhanced the ability of proliferation of testicular tissue cells in oligozoospermic mouse models (Figure 3(a)). These data demonstrated that SJD played promoting roles in the self-renewal, proliferation, and differentiation processes during which diploid SSCs produce haploid spermatozoa.

SSCs are the most primitive spermatogonia in the testis, and they play an essential role in maintaining highly productive spermatogenesis by self-renewal and continuous generation of daughter spermatogonia that differentiate into spermatozoa, transmitting genetic information to the next generation. Self-renewal- and proliferation-associated genes such as *Pou5f1*, *Lin28a*, *Mir-21*, *Mir-17∼92* (*Mirc1*), *Foxo1*, *Nanos2*, *Neurog 3*(*Ngn3*), *Sox3*, *Taf4b*, and *Zbtb16* (*Plzf*) maintain the capacity for self-renewal and proliferation of SSCs and progenitor spermatogonia [7–17]. During spermatogonial differentiation, the undifferentiated spermatogonia downregulate the self-renewal-associated genes and upregulate genes associated with differentiation such as *Sohlh1*, *Sohlh2*, *Stra8*, *Kit*, *Ccnd2,* and *Sall4* to promote SSCs to differentiate into mature haploid spermatozoa [18–23]. Our results indicated that SJD promoted spermatogenesis by upregulating the proliferation-associated gene *Lin28a* and differentiation-associated genes *Kit*, *Sohlh2,* and *Stra8* (Figure 3). Although the mechanisms are different between SJD and cyclophosphamide, SJD can partially antagonize the killing effect of cyclophosphamide on reproductive system cells. In fact, the causes of spontaneous male infertility are extremely complex [5, 46]. The impairment of spermatogenesis is the most common cause of male infertility [4]. SJD may play a more effective therapeutic role in clinical practice.

Sperm motility is an important factor involved in the overall sperm quality and is critical for ensuring fertilization success [47]. The mitochondrion is the major energy provider that sustains sperm motility. A previous study analyzed the effect of MMP on the temporal regulation of sperm motility. MMP can be considered as a potential regulator and indicator of sperm motility and, hence, could be directly related to male fertility [48–50]. Our data showed that SJD partly restored MMP levels of sperm impaired by cyclophosphamide inducing (Figures 4(a) and 4(b)). These data demonstrated that SJD played an important role in sustaining mitochondrial function and sperm motility. The membrane serves as a dynamic platform for the localization of various components that actively participate in all aspects of the motility process, including force generation, adhesion, signaling, and regulation [51]. Sperm plasma membrane integrity plays a crucial role in sperm motility and fertilization [52, 53]. Our results showed that sperm plasma membrane integrity was less impaired in oligozoospermic mouse models by SJD treatment than in those without SJD treatment (Figures 4(c) and 4(d)). These data provided strong evidence that SJD was an important factor involved in supporting sperm plasma membrane integrity and function.

## 5. Conclusions

SJD is a TCM prescription widely used to treat male infertility in many Chinese hospitals. It can increase sperm concentration, sperm vitality, sperm motility, and normal sperm morphology rate, to support sperm quantity and quality. SJD also promotes spermatogenesis and further demonstrates that it upregulates the proliferation-associated gene *Lin28a* and differentiation-associated genes *Kit*, *Sohlh2,* and *Stra8.* In addition, our data confirm that SJD increases sperm motility by sustaining MMP and sperm plasma membrane integrity.

## Data Availability

The data used to support the findings of this study are included within the article.

## Supplementary Materials

Supplementary Materials Supplemental information includes the details of all herbs used in SJD.
