# Supplementary material for: Sheng Jing Decoction Can Promote Spermatogenesis and Increase Sperm Motility of the Oligozoospermia Mouse Model
Source: Evid Based Complement Alternat Med. 2021 Dec 1;2021:3686494. doi: 10.1155/2021/3686494 (PMC8654543; doi:10.1155/2021/3686494)
Supplement: Supplementary Materials — Supplemental information includes the details of all herbs used in SJD. [file 3686494.f1.pdf]

SUPPLEMENTAL TABLE 1. The information of herbs in Sheng Jing Decoction

Official name	Local name	English name	Batch number	Producing area
Di Huang	Di Huang	*Rehmannia glutinosa* (Gaertn.) DC.	190323-2	Gansu, China
Huang Qi	Huang Qi	*Astragalus membranaceus* (Fisch.) Bunge	190326-2	Gansu, China
Tai Zi Shen	Tai Zi Shen	*Pseudostellaria heterophylla* (Miq.) Pax	190505-1	Guizhou, China
Xu Duan	Xu Duan	*Dipsacus acaulis* (A.Rich.) Napper	190320-1	Sichuan, China
Gou Qi Zi	Gou Qi	*Lycium arenicolum* Miers	190325-1	Ningxia, China
Sha Yuan Zi	Sha Yuan Zi	*Astragalus complanatus* Bunge	190109-1	Shanxi, China
Zao Jiao Ci	Zao Jiao Ci	*Gleditsia sinensis* Lam.	190304-1	Jiangsu, China
